# Systemic Injection of Oncolytic Vaccinia Virus Suppresses Primary Tumor Growth and Lung Metastasis in Metastatic Renal Cell Carcinoma by Remodeling Tumor Microenvironment

**DOI:** 10.3390/biomedicines10010173

**Published:** 2022-01-14

**Authors:** Jee Soo Park, Myung Eun Lee, Won Sik Jang, Jongchan Kim, Se Mi Park, Keunhee Oh, Namhee Lee, Won Sik Ham

**Affiliations:** 1 Department of Urology, Urological Science Institute, College of Medicine, Yonsei University, Seoul 03722, Korea; jsparkysmed@gmail.com (J.S.P.); lme0228@yuhs.ac (M.E.L.); sindakjang@yuhs.ac (W.S.J.); lumpakcef@yuhs.ac (J.K.); mistrie694@yuhs.ac (S.M.P.); 2 Department of Urology, Sorokdo National Hospital, Goheung 59562, Korea; 3 Department of Urology, Yongin Severance Hospital, Yonsei University Health System, Seoul 03722, Korea; 4 Research Center, SillaJen, Inc., Seoul 07325, Korea; khoh@kr.sillajen.com (K.O.); nhlee@kr.sillajen.com (N.L.)

**Keywords:** immunotherapy, oncolytic vaccinia virus, renal cell carcinoma, pexastimogene devacirepvec (pexa-vec), sunitinib

## Abstract

Immune checkpoint inhibitors and tyrosine kinase inhibitors are the first-line treatment for metastatic renal cell carcinoma (mRCC), but their benefits are limited to specific patient subsets. Here, we aimed to evaluate the therapeutic efficacy of JX-594 (pexastimogene devacirepvec, Pexa-vec) monotherapy by systemic injection in comparison with sunitinib monotherapy in metastatic orthotopic RCC murine models. Two highly metastatic orthotopic RCC models were developed to compare the treatment efficacy in the International Metastatic RCC Database Consortium favorable-risk and intermediate- or poor-risk groups. JX-594 was systemically injected through the peritoneum, whereas sunitinib was orally administered. Post-treatment, tumor microenvironment (TME) remodeling was determined using immunofluorescence analysis. Systemic JX-594 monotherapy injection demonstrated therapeutic benefit in both early- and advanced-stage mRCC models. Sunitinib monotherapy significantly reduced the primary tumor burden and number of lung metastases in the early-stage, but not in the advanced-stage mRCC model. Systemic JX-594 delivery remodeled the primary TME and lung metastatic sites by increasing tumor-infiltrating CD4/8+ T cells and dendritic cells. Systemic JX-594 monotherapy demonstrated significantly better therapeutic outcomes compared with sunitinib monotherapy in both early- and advanced-stage mRCCs by converting cold tumors into hot tumors. Sunitinib monotherapy effectively suppressed primary tumor growth and lung metastasis in early-stage mRCC.

## 1. Introduction

Approximately one-third of all patients diagnosed with renal cell carcinoma (RCC) already have systemic diseases at the time of the first diagnosis [1]. Although significant improvements in the therapeutic strategies for metastatic RCC (mRCC) have been achieved, the five-year survival rate is less than 10%, with incidence and mortality rates rising at a rate of 2–3% per decade [2]. mRCC remains a significant health issue as it is an incurable disease [3]. In the last decade, sunitinib (Sutent; Pfizer, New York, NY, USA), a multikinase inhibitor with antiangiogenic properties, has developed into a standard first-line treatment option for advanced mRCC [4]. However, cancer immunotherapy, particularly with immune checkpoint inhibitors (ICIs), has recently emerged as a potent and effective therapeutic strategy for advanced cancers, replacing sunitinib [5,6]. CheckMate 214 demonstrated the superiority of nivolumab and ipilimumab over sunitinib in patients with intermediate- and poor-risk disease [7]. After this study, immuno-oncology (IO) agents have changed the treatment paradigm for mRCC as IO combination therapies have now become the first-line therapy for mRCC [8].

Antibodies targeting immune checkpoints, such as cytotoxic T-lymphocyte associated protein-4, programmed cell death protein 1 (PD-1), and programmed death-ligand (PD-L1), demonstrate anti-tumor effects in various malignancies. However, ICI efficacy is limited in certain circumstances, deriving benefits for only a subset of patients. There is also a risk of primary refractory status and subsequent resistance and recurrence after IO combination therapies [9]. Moreover, IO combination therapy has no clear advantage over vascular endothelial growth factor receptor-tyrosine kinase inhibitors in the International Metastatic Renal Cell Carcinoma Database Consortium (IMDC) risk group [7,10,11].

The efficacy of ICIs is limited in the treatment of patients with advanced mRCC, with only 20–30% of patients responding to ICI monotherapy and others showing intrinsic resistance to ICI treatment due to a non-inflamed “cold” tumor microenvironment (TME) [12]. Thus, novel immunotherapeutic agents are warranted to overcome this limitation by changing the TME through the depletion of cancer-promoting microenvironmental cells and their reeducation toward immune-stimulating, tumor-suppressive phenotypes [13]. This strategy is now widely appreciated as an anticancer armamentarium [13,14]. Oncolytic virus (OV) is one of the most promising treatment strategies for solid malignancies [15] because it can remodel the TME toward a T cell-inflamed phenotype by stimulating host immune responses against the tumor [12].

JX-594 (pexastimogene devacirepvec, Pexa-vec) is a thymidine kinase-deleted oncolytic vaccinia virus and has been engineered to express granulocyte macrophage-colony stimulating factor (GM-CSF), an immune-activating transgene [16,17]. JX-594 possesses anticancer activity with low toxicity, as reported in preclinical and clinical studies [17]. Numerous clinical trials showed that JX-594 is one of the most feasible and promising OV platforms, along with a few OVs [16,17,18,19]. Few studies have demonstrated the immune-modulatory functions in the primary site of the TME after the intratumoral injection of JX-594 [17]. However, no studies have evaluated the therapeutic effects of JX-594 after its systemic injection in mRCC settings.

In this study, we aimed to evaluate the therapeutic efficacy of JX-594 monotherapy via systemic injection in comparison with sunitinib monotherapy in metastatic orthotopic RCC murine models. We developed two different models among metastatic orthotopic RCC murine models to compare the therapeutic efficacy according to different IMDC risk groups (favorable vs. intermediate or poor). Lastly, we demonstrated the dynamic remodeling of the TME in the primary tumor site as well as distant metastatic sites after systemic treatment with JX-594.

## 2. Materials and Methods

### 2.1. RCC Cell Lines

Three human RCC cell lines which consist of 786-O (cat# CRL-1932), Caki-1 (cat# HTB-46), A-498 (cat# HTB-44), ACHN (cat# CRL-1611), and Renca, the murine RCC cell line, were purchased from the American Type Culture Collection (Manassas, VA, USA). Roswell Park Memorial Institute 1640 medium or Eagle’s minimum essential medium in addition of 10% fetal bovine serum (Gibco; Thermo Fisher Scientific, Waltham, MA, USA) and 1% penicillin–streptomycin (Sigma-Aldrich, St. Louis, MO, USA) was used to culture cells (37 °C in a humidified atmosphere containing 5% CO_2_).

### 2.2. Treatment Regimens

JX-594 and a mouse variant of JX-594 (mJX-594) were provided by SillaJen, Inc. (Seoul, Korea). JX-594 is a Wyeth strain vaccinia virus modified by insertion of the human GM-CSF and Lac-Z genes into the vaccinia thymidine kinase gene region under the control of the synthetic early/late and p7.5 promoters, respectively [20]. mJX-594 is a Western Reserve strain of vaccinia virus encoding murine GM-CSF in the vaccina thymidine kinase gene locus under the control of the p7.5 promoter [21]. The virus was stored at −80 °C. The anticancer drug sunitinib was purchased from Selleckchem (Houston, TX, USA).

### 2.3. Cell Proliferation (Cytotoxicity) Assay

Cells were seeded in 96-well culture plates at a density of 1 × 10^4^ cells per well. The next day, the cells were treated with JX-594 or mJX-594 at the desired concentration in culture media. At 72 h after treatment, cell viability was assessed using CCK-8 assay (Dojindo Laboratories, Kumamoto, Japan) in accordance with the manufacturer’s instructions. Absorbance was detected at 450 nm using the VersaMax microplate reader (Molecular Devices, Sunnyvale, CA, USA).

### 2.4. Cell Cycle Analysis

Cells were treated with mJX-594 for 24 h. To analyze the cell cycle, treated cells were harvested, washed in phosphate-buffered saline (PBS), resuspended in ice-cold 70% ethanol, and then incubated overnight. Ethanol was removed, and cells were washed with PBS prior to staining with PI/RNase staining buffer (BD Pharmingen, Franklin Lakes, NJ, USA). Cells were incubated for 15 min at room temperature and analyzed on a BD LSR II cell analyzer (BD Biosciences, San Jose, CA, USA). Cell cycle modeling was performed using FlowJo v10 software (FlowJo LLC, Ashland, OR, USA).

### 2.5. TUNEL, Migration, and Invasion Assay

For apoptotic cell analysis, PBS- or mJX-594-treated cells were washed with PBS, fixed in 4% paraformaldehyde for 15 min at room temperature, and permeabilized with 0.3% Triton X-100 for 5 min. Apoptotic cells with DNA breaks were detected using the In Situ Cell Death Detection Kit, TMR red (#12 156 792 910; Roche, Indianapolis, IN, USA) according to the supplier’s protocol. For migration assay, cells were treated with PBS or mJX-594 (1 MOI) and plated in the top chamber (5 × 10^4^ cells/well) of 24-well Transwell (8.0 μm pore size; Corning Inc., Corning, NY, USA). Then, a 10% fetal bovine serum-containing medium was placed in the bottom Transwell chamber, and the assembly was incubated at 37 °C for 24 h. In vitro Matrigel invasion assays were performed using CytoSelect™ 24-Well Cell Invasion Assay kit (Cell Biolabs, San Diego, CA, USA) following the manufacturer’s manual. Migrating or invading cells on the bottom surface of the membrane were stained with crystal violet and quantified at OD 560 nm after extraction using the VersaMax microplate reader (Molecular Devices, Sunnyvale, CA, USA).

### 2.6. Tumor Models and Treatments

The protocol was approved by the Institutional Animal Care and Use Committee (IACUC) of the Yonsei University Health System (IACUC No. 2020-0006) which follows the guidelines specified by the Institute of Laboratory Animal Resources Commission on Life Sciences National Research Council in the USA. All animal experiments were conducted following the Guide for the Care and Use of Laboratory Animals.

**Figure 1:**
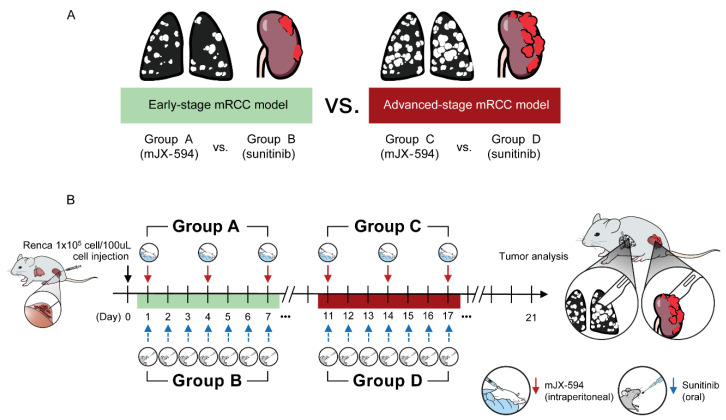
Two pulmonary metastatic orthotopic renal cell carcinoma murine models. (**A**) An early-stage metastatic renal cell carcinoma (mRCC) model was used to compare groups A (mJX-594 monotherapy) and B (sunitinib monotherapy). The advanced-stage mRCC model was used to compare groups C (mJX-594 monotherapy) and D (sunitinib monotherapy). (**B**) In vivo selected tumor cells from pulmonary metastases for reimplanted (1 × 10^5^ cells/100 μL) and treatments started on the first day of implantation for groups A and B. For groups C and D, treatments started on the 11th day of implantation. mJX-594 was intraperitoneally injected every 3 days for three times, whereas sunitinib was treated by gavage daily for 7 days. After the 21st day of injection, analysis was performed.

Adult male BALB/c mice (Orient Bio Inc., Seongnam, GyeongGi-Do, Korea) aged 6–7 weeks were used in this study. We used highly pulmonary orthotopic RCC murine models that were previously developed by our team [22]. To develop a clinically relevant murine RCC model that exhibited enhanced pulmonary metastasis, mice underwent intrarenal implantation using Renca cells (1 × 10^5^ cells/100 μL), and an in vivo selection of tumor cells from pulmonary metastases for reimplantation was performed, which is described elsewhere in detail [22]. Using this model, two models were developed (Figure 1). The first was the early-stage mRCC model, which started receiving the treatment either by mJX-594 (group A) or sunitinib (group B) after the 1st day of implantation of in vivo selected tumor cells. The second was the advanced-stage mRCC model, which started receiving the treatment either by mJX-594 (group C) or sunitinib (group D) after the 11th day of implantation of in vivo selected tumor cells. mJX-594 (1 × 10^7^ plaque-forming units [pfu] per mouse) was intraperitoneally injected every 3 days for three times. The mice in the control group were treated with the same volume of mJX-594 for PBS every 3 days for three times. For the experiments of angiogenesis inhibitors, the mice received sunitinib (40 mg/kg in 5 µL/g sterile saline with 0.5% methylcellulose suspension per mouse) by gavage daily for 7 days. After the 21st day (3 weeks) of injection, the mice were sacrificed, and their kidney tissues were harvested and weighed. Their lungs were inflated with India ink to visualize lung tumor nodules.

### 2.7. IFN-ɣ Enzyme-Linked Immunospot (ELISPOT) Assay

For measurement of tumor-specific cytotoxic T cells, splenocytes and tumor-infiltrating lymphocytes (TILs) from each group were isolated 3 days after the final treatments. Cells were labeled with anti-CD8a microbeads (Miltenyi Biotec, Auburn, CA, USA), then purified using MACS (Miltenyi Biotec). CD8+ T cells were enriched to more than 90% purity and purified CD8+ T cells were co-cultured with Renca tumor cells (10:1 ratio) in 96-well plates which are precoated with mouse IFN-ɣ (Mabtech, Inc., Cincinnati, OH, USA). Plates were incubated with 1 μg/mL of the biotinylated anti-mouse IFN-ɣ antibody R4-6A2-biotin for 2 h at room temperature then addition and incubation of streptavidin-ALP solution for 1 h at room temperature. Finally, spots were analyzed using ImageJ software after the addition of BCIP/NBT-plus substrate solution.

### 2.8. Immunofluorescence Staining

Immunofluorescence staining was performed in the advanced-stage mRCC model since significant therapeutic efficacy of JX-594 monotherapy was observed in the advanced-stage model. Tissues were fixed in 10% formalin overnight and then transferred to 70% ethanol. The samples were paraffin-embedded, sectioned, and stained using the following primary antibodies: rabbit anti-CD31 (EPR17260-263; Abcam, Cambridge, MA, USA), rat anti-CD8 (YTS169.4; Abcam), rat anti-CD4 (RM4-5; BD Pharmingen), rabbit anti-PD-L1 (EPR20529; Abcam), mouse anti-cytokeratin (C11; Santa Cruz Biotechnology, Santa Cruz, CA, USA), rat anti-Foxp3 (FJK-16s; Invitrogen, Waltham, MA, USA), hamster anti-CD11c (HL3; BD Pharmingen), and rabbit anti-VV (Abcam). After washing, the slides were incubated with the following secondary antibodies: FITC-conjugated or Texas Red-conjugated anti-rabbit IgG (Vector Laboratories, Burlingame, CA, USA), FITC-conjugated anti-rat IgG (Jackson ImmunoResearch, West Grove, PA, USA), Texas Red-conjugated anti-mouse IgG (Vector Laboratories), or FITC-conjugated anti-hamster IgG (Jackson ImmunoResearch). Finally, the samples were mounted with VECTASHIELD^®^ Mounting Medium (Vector Laboratories). The immunofluorescence images were captured using the Zeiss LSM700 confocal microscope (Carl Zeiss Microscopy GmbH, Jena, Germany). Staining was quantified using the ImageJ software. Signal intensity was calculated as the number of positive-staining pixels relative to the total number of pixels per tumor section (% positive).

### 2.9. Statistical Analysis

Statistical analyses were performed using GraphPad Prism version 8.0 (GraphPad Software, Inc., La Jolla, CA, USA) and SPSS version 23.0 (IBM Corp., Armonk, NY, USA). All results were expressed as the mean ± standard deviation (s.d.) unless otherwise indicated. Student’s *t*-test was used unless the dataset did not follow a normal distribution on a Shapiro–Wilk normality test. If the dataset did not follow a normal distribution, the Mann–Whitney U test was used. All statistical tests were two-tailed, and *p*-values < 0.05 were considered significant.

## 3. Results

### 3.1. Effect of JX-594 in Human and Murine RCC Cell Lines Resulting in Cytotoxicity, Cell Cycle Arrest, and Decrease in Migration and Invasion Ability

**Figure 2:**
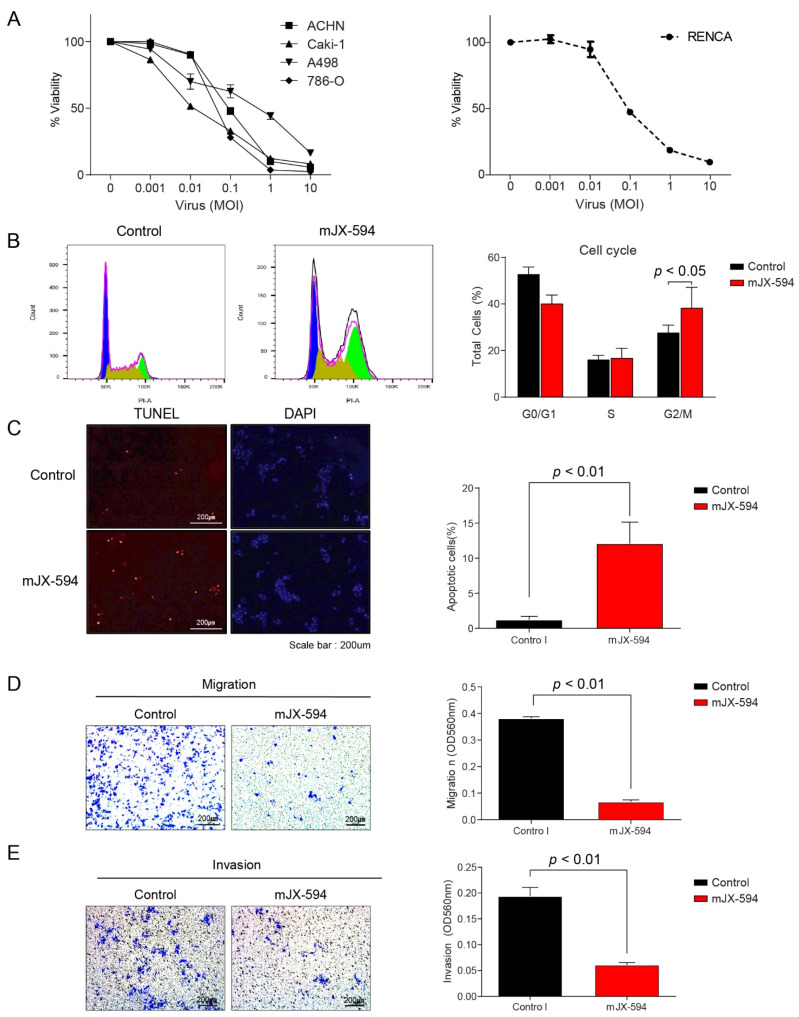
JX-594 has a direct oncolytic effect against human and murine renal cell carcinoma (RCC). (**A**) Cell viability of human (786-O, A498, ACHN, Caki-1) and murine (Renca) RCC cell lines treated with escalating doses of JX-594 or mJX-594. (**B**) Cell cycle analyses of mJX-594-treated cells. Renca cells treated with mJX-594 (1 MOI) were analyzed by flow cytometry for DNA content at 24 h post-treatment. Representative cell cycle histograms (left) and quantification of cell cycle distribution (right). (**C**) Apoptosis of Renca cells treated with mJX-594 or control (PBS) was analyzed using the TUNEL assay; TUNEL-positive cells (pseudocolored red), nuclei (pseudocolored blue), scale bar: 200 μm. (**D**) Migration of Renca cells was evaluated and compared after the treatment with mJX-594 or control (PBS), scale bar: 200 μm. (**E**) Invasion of Renca cells was evaluated and compared after the treatment with mJX-594 or control (PBS), scale bar: 200 μm. Values are mean ± SD. PBS, phosphate-buffered saline. All experiments were conducted at least three times.

Treatment of 786-O, Caki-1, A-498, and ACHN with JX-594 resulted in a dose-dependent decrease in cell viability at 72 h post-treatment (Figure 2A). Similar to the human RCC cell lines, infection of Renca cells with mJX-594 resulted in cytotoxicity (Figure 2A). Following treatment of mJX-594, Renca cells resulted in a significant increase in G2/M-phase cells (38.6 ± 8.5% vs. 27.9 ± 3.0%, *p* < 0.05), known as G2/M-phase arrest (Figure 2B). Terminal deoxynucleotidyl transferase-mediated dUTP nick-end labeling (TUNEL) assay revealed significantly increased apoptosis (*p* < 0.01; Figure 2C). The migration ability of Renca cells decreased after treatment with mJX-594, with a significantly lower repair with the starch test compared with that in the control group (*p* < 0.01; Figure 2D). The number of invasive Renca cells penetrating the membrane after treatment for 24 h with mJX-594 was significantly lower compared with that in the control group (*p* < 0.01; Figure 2E).

### 3.2. Comparison of Therapeutic Efficacy of JX-594 and Sunitinib in Early- and Advanced-Stage mRCC

**Figure 3:**
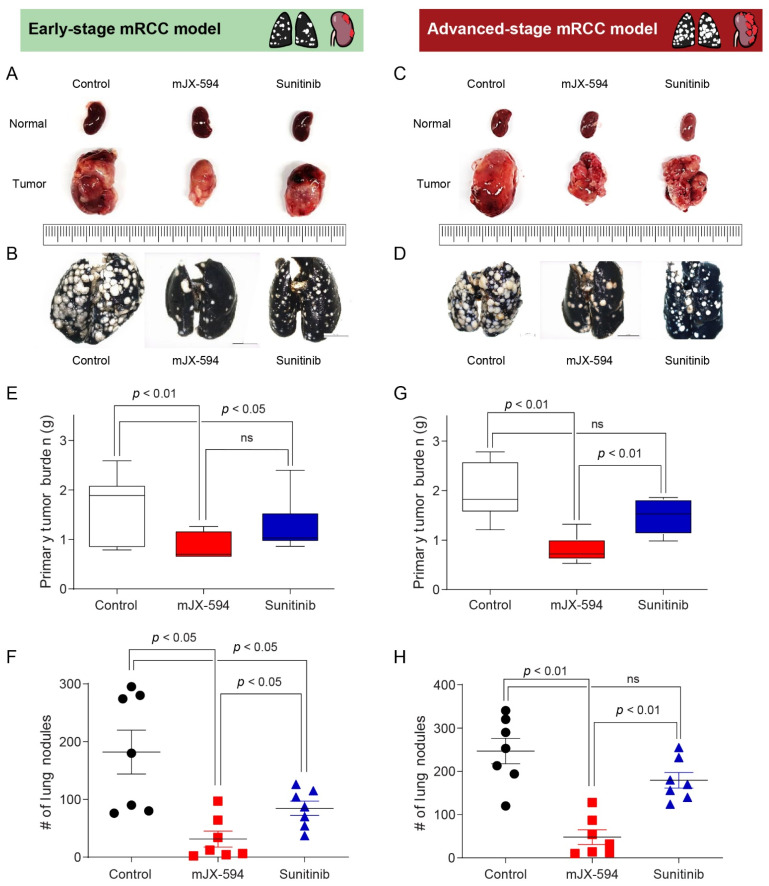
Representative images and comparisons of primary tumor burden and number of lung nodules in mJX-594-treated, sunitinib-treated, and control (PBS-treated) mice. (**A**) Comparison of primary tumor burden after the treatments in early-stage metastatic renal cell carcinoma (mRCC) model. (**B**) Comparison of lung metastasis after the treatments in early-stage mRCC model. (**C**) Comparison of primary tumor burden after the treatments in advanced-stage mRCC model. (**D**) Comparison of lung metastasis after the treatments in advanced-stage mRCC model. Therapeutic efficacy of mJX-594 and sunitinib treatment in early- and advanced-stage mRCC models. (**E**) Comparison of primary tumor burden after the treatments with mJX-594, sunitinib, or control (PBS) in early-stage mRCC model. (**F**) Comparison of lung metastasis after the treatments with mJX-594, sunitinib, or control (PBS) in early-stage mRCC model. (**G**) Comparison of primary tumor burden after the treatments with mJX-594, sunitinib, or control (PBS) in advanced-stage mRCC model. (**H**) Comparison of lung metastasis after the treatments with mJX-594, sunitinib, or control (PBS) in advanced-stage mRCC model. Values are mean ± SD. PBS, phosphate-buffered saline; ns, not significant; #, number.

To demonstrate and compare the therapeutic efficacy of mJX-594 or sunitinib monotherapy between the favorable-risk group and the intermediate- or poor-risk group according to the IMDC criteria, we developed two models (Figure 1 and Table 1). The early-stage model was designed to resemble the favorable-risk group, and the advanced-stage model reflected the intermediate or poor-risk group (Figure 1). mJX-594 and sunitinib treatments reduced the primary tumor burden (Figure 3A) and metastatic lung lesions (Figure 3B) in early-stage mRCC. In advanced-stage mRCC, mJX-594 treatment reduced the primary tumor burden (Figure 3C) and metastatic lung lesions (Figure 3D), but no therapeutic effects were observed with sunitinib monotherapy.

In early-stage mRCC, mJX-594 treatment significantly reduced both the primary tumor burden (*p* < 0.01) and metastatic lung lesions (*p* < 0.05) than did the control (Figure 3E,F). Similarly, sunitinib treatment significantly reduced the primary tumor burden (*p* < 0.05) and metastatic lung lesions (*p* < 0.05) than did the control (Figure 3E,F). Regarding the therapeutic efficacy for primary tumor lesions, no statistical differences were observed between the mJX-594 and sunitinib treatments (Figure 3E). In terms of the therapeutic efficacy for metastatic lung lesions, mJX-594 treatment demonstrated significantly better outcomes than those of sunitinib treatment (*p* < 0.05) (Figure 3F).

In advanced-stage mRCC, mJX-594 treatment significantly reduced the primary tumor burden (*p* < 0.01) and metastatic lung lesions (*p* < 0.01) than did the control (Figure 3G,H). Sunitinib treatment also reduced the primary tumor burden and metastatic lung lesions than did the control, but no statistical differences were found. mJX-594 treatment demonstrated superior efficacy compared with sunitinib treatment in terms of the primary tumor burden and metastatic lung lesion reduction in advanced-stage mRCC.

### 3.3. JX-594 Systemic Treatment Increases Local Cancer-Specific Immune Responses by Converting Immunosuppressive Noninflamed Tumors into Inflamed Tumors in Primary Tumor

To determine whether systemic injection of JX-594 could induce both primary tumor and distant lung metastatic site immune responses, we administered mJX-594 into the peritoneum. Systemic injection of mJX-594 was generally well-tolerated without significant weight loss, and no treatment-related mortalities were observed (Supplementary Materials Figure S1).

**Figure 4:**
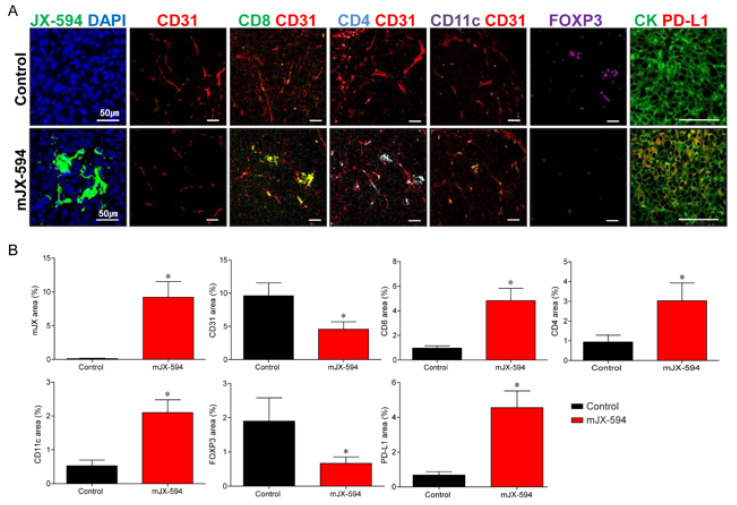
mJX-594 systemic treatment activates anti-cancer immunity by converting immunosuppressive noninflamed tumors into inflamed tumors at primary tumor. (**A**) Representative images of primary tumors treated with mJX-594 systemic treatment. Tumor sections were stained for JX-594, CD31, CD8, CD4, CD11c, Foxp3, and PD-L1. Scale bars, 50 μm. (**B**) Quantifications of the JX-594+ area, CD31+ blood vessels, CD8+ T cells, CD4+ T cells, CD11c+ dendritic cells, Foxp3+ regulatory T cells, and PD-L1+ cells. Values are mean ± SD. * *p* < 0.05 vs. control. 6 animals per group were used.

To determine the immunomodulatory potential of JX-594 in the therapeutic efficacy of primary tumor, changes in the TME of the primary tumor after systemic treatment of mJX-594 were evaluated (Figure 4). The tumoral level of mJX-594 was significantly increased, whereas tumor vessel density was significantly reduced after the mJX-594 treatment. The population of CD8+ cytotoxic T cells within the tumor, which comprise the most critical aspect of anticancer immunity, increased after the mJX-594 treatment. CD4+ T cells and CD11c+ dendritic cells (DCs) also increased after the mJX-594 treatment. The number of Foxp3+ regulatory T cells (Treg) reduced after the mJX-594 treatment. Conversely, the inhibitory checkpoint molecule, PD-L1, significantly increased. Collectively, noninflamed tumors were converted into T cell-inflamed tumors by JX-594 systemic treatment.

### 3.4. Systemic Injection of JX-594 Leads to Distant Lung Metastatic Sites Cancer-Specific Immune Responses

**Figure 5:**
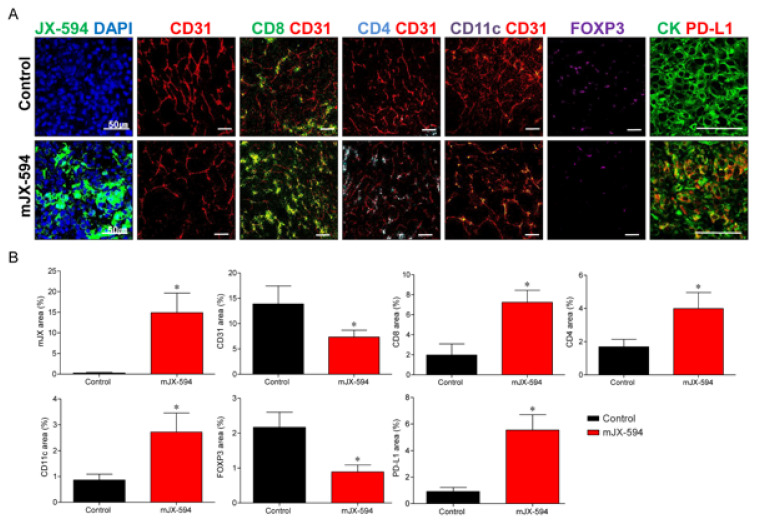
mJX-594 systemic treatment activates anti-cancer immunity by converting immunosuppressive noninflamed tumors into inflamed tumors at lung metastatic sites. (**A**) Representative images of lung metastatic sites treated with mJX-594 systemic treatment. Tumor sections were stained for JX-594, CD31, CD8, CD4, CD11c, Foxp3, and PD-L1. Scale bars, 50 μm. (**B**) Quantifications of the JX-594+ area, CD31+ blood vessels, CD8+ T cells, CD4+ T cells, CD11c+ dendritic cells, Foxp3+ regulatory T cells, and PD-L1+ cells. Values are mean ± SD. * *p* < 0.05 vs. control. 6 animals per group were used.

Immunofluorescence analyses revealed similar alterations in the innate and adaptive immunity in the lung metastatic sites (Figure 5 and Figure S2 (high-magnification version)), which was demonstrated in the primary tumor. Regarding innate immunity, mJX-594 treatment significantly increased the number of DCs 4.2-fold (*p* < 0.001). Similarly, mJX-594 treatment increased the adaptive immunity by increasing the number of CD8+ cytotoxic T cells 4.8-fold and that of CD4+ T cells 3.3-fold compared with those of the control group, with statistical significance (*p* < 0.001). Collectively, these findings indicate that JX-594 treatment effectively suppressed the progression of lung metastatic lesions via enhanced innate and adaptive immunity.

### 3.5. Comparison of Changes in TME between Primary Tumor and Lung Metastatic Sites

Changes in the TME between the primary tumor and lung metastatic sites were compared (Table 2). mJX-594 increased 49.7- and 92.0-fold in the primary tumor and lung metastatic sites, respectively, demonstrating more significant increases in the tumor level of JX-594 in lung metastatic sites (*p* = 0.028). Increases in terms of fold changes in CD4/CD8+ T cells and CD11c+ DCs in the lung metastatic sites were significantly higher than those in the primary tumor (CD8+ T cells: *p* = 0.019, CD4+ T cells: *p* = 0.004, CD11c+ DCs: *p* = 0.017). No significant differences were noted in terms of fold changes in the CD31+ blood vessel, Foxp3+ Treg, and PD-L1+ cells between the primary tumors and lung metastatic sites.

IFN-ɣ ELISPOT assays have shown a significant increase in IFNγ-secreting T cells against Renca tumor cells within primary tumors, lung metastatic sites, and spleens of JX-594 treated mice compared with control mice (Supplementary Materials Figure S3).

## 4. Discussion

In this study, we demonstrated that the systemic delivery of JX-594 monotherapy was an effective therapeutic strategy for mRCC, both reducing the primary tumor burden and lung metastatic sites without adverse reactions. Although the combination therapy with ICI was not within the scope of this study and was therefore not evaluated here, JX-594 monotherapy itself sufficiently converted noninflamed tumors to inflamed tumors, enabling the host immune system to eradicate the tumor cells. Our findings provide evidence that the oncolytic vaccinia virus, JX-594, is an effective therapeutic modality not only in early-stage mRCC but also in advanced-stage mRCC by changing the TME to stimulate host immune responses against the tumor. Sunitinib, which remains the first-line therapy for mRCC, has also exhibited therapeutic efficacy in early-stage mRCC both in the primary tumors and lung metastatic sites.

Viruses have evolved to evade the host immune responses after years of co-evolution with our immune systems [23]. With their evolution and the advancement of technology, viruses, namely OVs, have been therapeutically engineered to fight against cancers [12]. OVs selectively infect and kill the cancer cells without disrupting the normal cells [17,24]. They induce direct destruction of tumors through selective infection and tumor-cell lysis [6,25]. During this process, immunogenic cell death occurs with the widespread release of tumor-associated antigens [6]. These antigens are presented by the DCs to activate the antitumor immunity by inducing antigen-specific T-cell responses with the expansion of cytotoxic effector cells, eventually acting as an in situ cancer vaccine within the TME [26].

In our study, JX-594 effectively remodeled the TME both in the primary tumors (Figure 4) and distant lung metastatic sites (Figure 5) through the activation of the immune system, which increased the tumor-infiltrating CD4/8+ T cells and DCs. JX-594 is known for its tumor control ability by T cell-mediated mechanism rather than direct oncolysis [17]. Our study also demonstrated that the activation of antitumor immunity significantly induced the regression of the primary tumor burden and the number of lung metastases through T cell-mediated mechanism, demonstrating the therapeutical benefits of JX-594 systemic injection.

Few OVs have been evaluated in many preclinical and clinical studies, and talimogene laherparepvec (Imlygic) was the first oncolytic herpesvirus to be approved and clinically used for the treatment of advanced melanoma [27]. For JX-594, a phase Ib clinical trial (NCT03294083) addressed patients with mRCC treated with IV infusion of JX-594 combined with cemiplimab (anti-PD-1), which reported an overall response rate of 37.5% with an acceptable safety profile in an American Association for Cancer Research meeting [28].

JX-594 activated the anticancer immunity; however, the inhibitor checkpoint molecule, PD-L1, was increased, suggesting the induction of negative feedback mechanisms to counter-balance the immune response in the TME. A similar result was reported by Chon et al. wherein PD-L1 upregulation immediately followed a massive influx of CD8+ T cells [17]. Therefore, the combinatorial use of ICIs seems plausible considering the upregulated inhibitor checkpoint molecule by JX-594. Several studies have evaluated the therapeutic efficacy of combining OVs and ICIs [12]. Although the scope of this study was limited to testing the therapeutic efficacy of JX-594 monotherapy, the combination therapy of JX-594 and ICIs could be more potently effective, as shown in a previous study [6].

However, considering the therapeutic efficacy of JX-594 monotherapy, the need for combining ICIs requires reconsideration. Although some of the combination strategies have demonstrated benefits compared with monotherapy, we should not overlook that combination therapy might lead to overtreatment, sparing the unnecessary toxicity and financial burden in cases that would have shown equivalent benefits from monotherapy. ICIs risk causing immune-mediated adverse effects, such as skin rash, pruritus, pneumonitis, diarrhea and/or immune-mediated colitis, hepatitis, and problems in the endocrine system [29]. A recent review article by Schirrmacher [30] reported that OVs exert profoundly lower side effects, including major adverse events (WHO grades 3–4), than did other systemic therapies in patients with cancer. Therefore, considering the sufficient therapeutic effects of JX-594, its use as monotherapy should also be considered.

The major challenge of standardizing OV is the proper delivery of viruses to the tumor site and eliciting antitumor immunity. To achieve significant efficacy in patients with cancer, OVs need to be delivered intratumorally [31]. Low efficiencies of both viral delivery to the tumor tissues and viral replication throughout the entire tumor tissue remain a challenge. These properties severely restrict the magnitude of therapeutic efficacy. Another hurdle is that the TME is highly immunosuppressive, and most OVs may not be able to modulate this effectively into pro-antitumor immunity [31]. The intratumoral injection of mJX-594 obviously induces a local immune response [17]. However, whether the systemic delivery of JX-594 could induce changes in the TME both in the primary tumors and the lung metastatic sites was uncertain. In this study, we showed that the systemic injection of JX-594 was well-delivered to the primary tumor and lung metastatic sites. Interestingly, the tumoral level of JX-594 increased more in the lung metastatic sites compared with that in the primary tumors. Although the mechanism is unclear, we believe that this is due to the characteristics of the vaccinia virus. OV has an inherent capability of selectively targeting tumors, which was intensified through viral thymidine kinase gene deletion that led to significant attenuation in normal tissues [32]. During the development of a metastatic orthotopic RCC murine model, bioluminescent imaging (BLI) showed that lung metastatic sites have higher BLI signal intensities than do primary tumors as the days pass after the injection, demonstrating that active cancer cells are more densely placed at the metastasis sites [22]. Although it is only a hypothesis, malignant tumors metastasize to escape to a friendlier environment where a higher payoff is initially possible [33]. If OVs are more reluctant to move to a friendlier environment for cancer cells as shown in our study, the value of the OVs will be much higher. Further studies are warranted to clarify this finding.

Sunitinib continues to be administered as the first-line therapeutic agent in mRCC [34]. The European Association of Urology Guidelines on RCC recommend that, in patients who cannot receive or tolerate immune checkpoint inhibition, monotherapies with sunitinib, pazopanib, and cabozantinib are alternative treatment options [35]. The NCCN Kidney Cancer Panel also lists sunitinib as a category 1 preferred option for the first-line treatment for mRCCs with good-risk features, while recommended as an other option in mRCCs with poor- or intermediate-risk features [36]. Our study also showed the robust therapeutic efficacy of sunitinib in the IMDC favorable-risk group, especially in reducing primary tumor burden. Furthermore, since second-line sunitinib demonstrates clinical activity after the failure of frontline immunotherapy in patients with mRCC, the role of TKIs should be reconsidered in this era where immunotherapy replaces targeted therapy [37].

While sunitinib is associated with a potent angiogenic activity, recent clinical studies have highlighted its immune-modulatory effects [38,39]. Lawson et al. reported that sunitinib augments the immunotherapeutic efficacy of reovirus [38]. Ongoing angiogenesis contributes to immune evasion through the induction of a highly immunosuppressive TME [39]. Therefore, the use of angiogenesis inhibitor sunitinib to promote leukocyte infiltration into the tumor is an effective strategy to improve oncolytic virotherapy, although some of the limitations have to be overcome due to the poor understanding of the interaction between immunity and the vasculature. Special considerations for scheduling, dosing, and sequencing between immunotherapy agents and anti-angiogenic agents should be made since they assert antitumor activities at different stages, and their spatial and temporal modes of action might differ [39].

As reported by Chon et al., the tumor vessel density also reduced after the mJX-594 treatment in our study [17]. Along with a previous study, we also demonstrated that JX-594 is a potent but transient tumor vessel disruptor. As previously mentioned, angiogenesis is an important immune evasion mechanism [39], and vessel disrupting effects might also contribute to tumor regression.

In this study, we developed two models based on the IMDC risk criteria. Although many treatment agents exist, these agents are recommended based on the IMDC risk criteria, which were developed in the TKI era [40]. These criteria are outdated considering the advancements in mRCC treatment. Therefore, future studies are needed to optimize the treatment strategies using specific molecular biomarkers to facilitate the clinical decision-making process [41].

Our study has its strength in evaluating the therapeutic efficacy of systemic treatment of JX-594 through two different models based on the IMDC risk criteria. Moreover, we have demonstrated the dynamic changes of TME in both primary TME and lung metastatic sites. However, we were not able to demonstrate the therapeutic efficacy of combination therapy with JX-594 and ICIs since it was not the original scope of this study. Future studies are planned to evaluate the therapeutic efficacy of combination therapy of JX-594 and ICIs in mRCC. Second, we have only evaluated the DCs for the innate immunity since the mechanism of OVs is associated with antigen presentation to DCs. However, since sunitinib is known to be associated with myeloid-derived suppressor cells (MDSCs) which have immunosuppressive and proangiogenic activities [42], we are currently under investigation of the role of sunitinib in the immunotherapy era by including MDSCs in future studies.

## 5. Conclusions

The systemic injection of JX-594 dynamically remodeled the TMEs from those of cold to those of hot tumors and activated anti-cancer immunity, which suppressed both the primary tumors and the lung metastases in early- and advanced-stage mRCCs. Sunitinib has limited efficacy in early-stage mRCC, but its role in improving the immunotherapeutic outcomes should be further investigated. Our study is the first to provide the findings that JX-594 monotherapy could be considered as the treatment option for mRCC although future clinical studies are needed.

## Figures and Tables

**Table 1:** Comparison of therapeutic efficacy of JX-594 and sunitinib monotherapy in early- and advanced-stage metastatic renal cell carcinoma models.

	Early-Stage		Advanced-Stage	
IMDC Criteria	Favorable-Risk Group		Intermediate- or Poor-Risk Group	
	Primary tumor	Metastatic sites	Primary tumor	Metastatic sites
JX-594	(+)	(+)	(+)	(+)
Sunitinib	(+)	(+)	(−)	(−)

(+) denotes superior therapeutic efficacy compared with the control group. (−) denotes no superior therapeutic efficacy compared with the control group.

**Table 2:** Comparison of therapeutic efficacy of JX-594 monotherapy in advanced-stage metastatic renal cell carcinoma models.

	Fold-Change (JX-594/Control)		*p*-Value
Primary tumor	mJX-594	49.7	<0.001
	CD31+ blood vessel	0.5	0.002
	CD8+ T cells	3.6	<0.001
	CD4+ T cells	2.4	<0.001
	CD11c+ DCs	3.0	<0.001
	Foxp3+ Treg	0.4	<0.001
	PD-L1+ cells	6.1	<0.001
Lung metastatic sites	mJX-594	92.0	<0.001
	CD31+ blood vessel	0.5	<0.001
	CD8+ T cells	4.8	<0.001
	CD4+ T cells	3.3	<0.001
	CD11c+ DCs	4.2	<0.001
	Foxp3+ Treg	0.4	0.002
	PD-L1+ cells	6.6	<0.001

*p*-value was calculated between JX-594 vs. control.

## Data Availability

The datasets used and/or analyzed in this study are available from the corresponding author upon reasonable request.

## Supplementary Materials

The following supporting information can be downloaded at: https://www.mdpi.com/article/10.3390/biomedicines10010173/s1, Figure S1: Mice weight over time, demonstrating no significant weight loss in JX-594 or sunitinib treatment group compared with control group according to (A) early-stage metastatic renal cell carcinoma (mRCC) model and (B) advanced-stage mRCC model; Figure S2: Representative high-magnified images of lung metastatic sites treated with mJX-594 systemic treatment. Tumor sections were stained for CD31, CD8, CD4, CD11c, Foxp3, and PD-L1. Scale bars, 10 μm.
