# Supplementary material for: Systemic Injection of Oncolytic Vaccinia Virus Suppresses Primary Tumor Growth and Lung Metastasis in Metastatic Renal Cell Carcinoma by Remodeling Tumor Microenvironment
Source: Biomedicines. 2022 Jan 14;10(1):173. doi: 10.3390/biomedicines10010173 (PMC8773601; doi:10.3390/biomedicines10010173)
Supplement: Supplementary file 1 [file biomedicines-10-00173-s001.zip › biomedicines-1464408-supplementary.pdf]

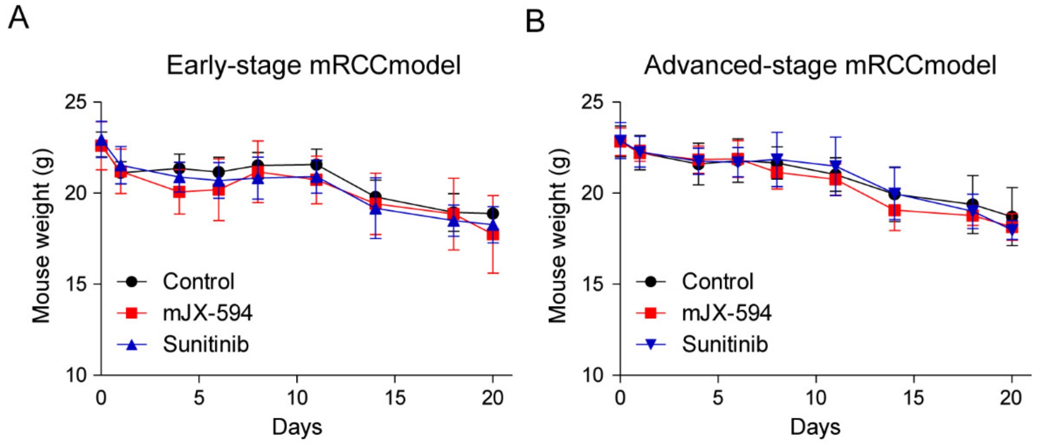

Supplementary Figure S1. Mice weight over time, demonstrating no significant weight loss in JX-594 or sunitinib treatment group compared with control group according to (A) early-stage metastatic renal cell carcinoma (mRCC) model and (B) advanced-stage mRCC model.

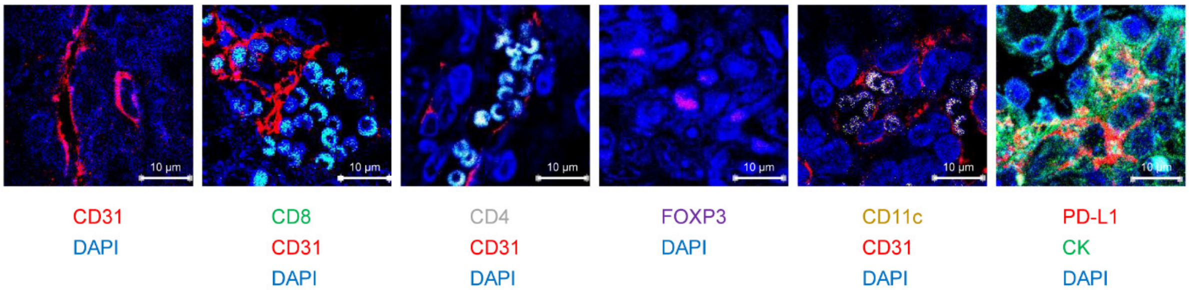

Supplementary Figure S2. Representative high-magnified images of lung metastatic sites treated with mJX-594 systemic treatment. Tumor sections were stained for CD31, CD8, CD4, CD11c, Foxp3, and PD-L1. Scale bars, 10 µm.

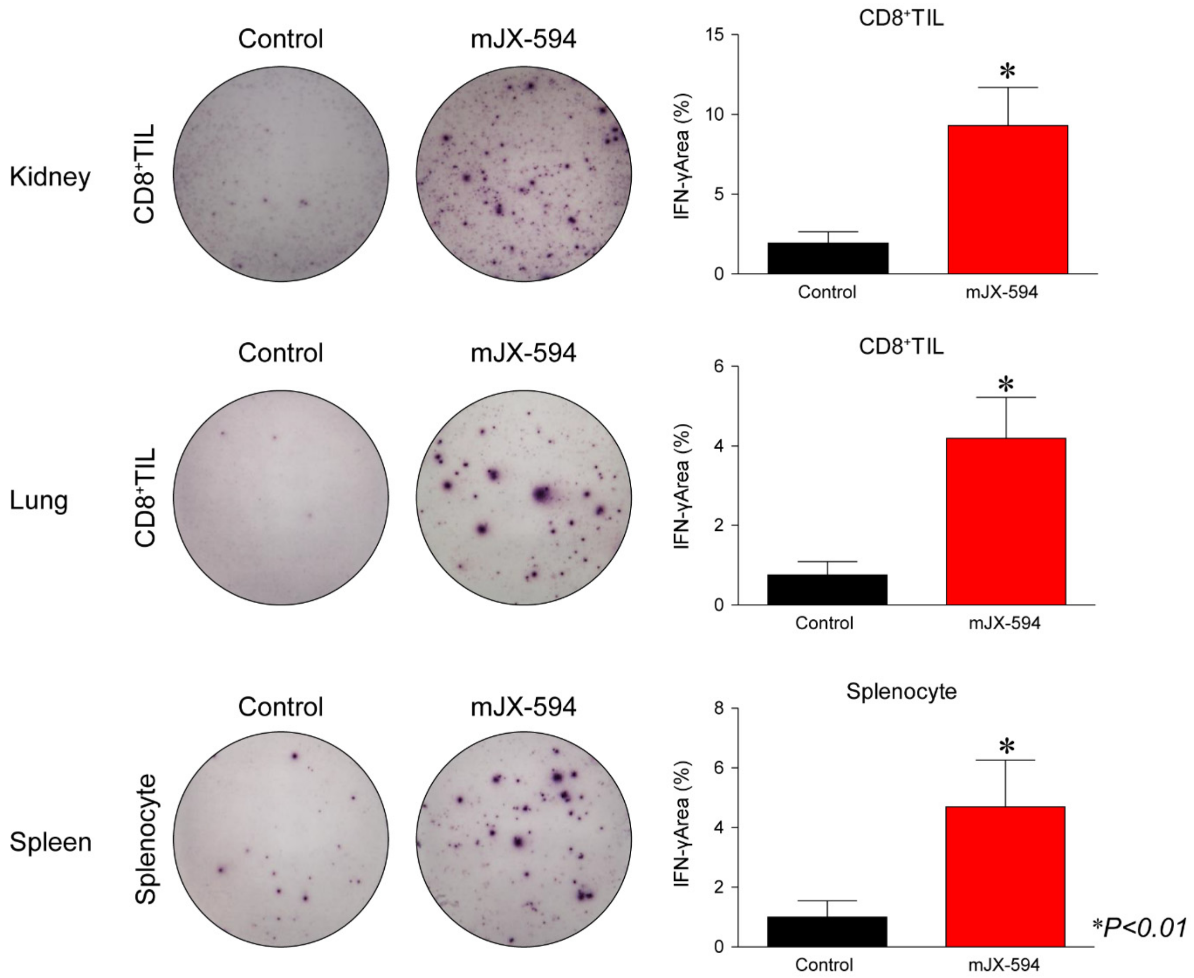

Supplementary Figure S3. Images and comparisons of IFNγ ELISPOT in CD8+ tumor infiltrating lymphocytes (TILs) and splenocytes from control or JX-594 treated mice. CD8+ TILs were isolated from dissociated tumors with anti-CD8 antibody-coated magnetic beads. * $P<0.01$
